# Reliability of rubidium-82 PET/CT for renal perfusion determination in healthy subjects

**DOI:** 10.1186/s12882-022-02962-w

**Published:** 2022-11-28

**Authors:** Stine Sundgaard Langaa, Frank Holden Mose, Claire Anne Fynbo, Jørn Theil, Jesper Nørgaard Bech

**Affiliations:** 1 University Clinic in Nephrology and Hypertension, Department of Medicine, Gødstrup Hospital and Aarhus University, Herning, Denmark; 2 Clinic for Nuclear Medicine, Gødstrup Hospital, Herning, Denmark; 3 Department of Clinical Medicine, Aarhus University, Aarhus, Denmark

**Keywords:** Rubidium-82, PET/CT, Pharmacokinetic modelling, Renal clearance, Renal blood Flow, Renal plasma flow

## Abstract

**Background:**

Changes in renal perfusion may play a pathophysiological role in hypertension and kidney disease, however to date, no method for renal blood flow (RBF) determination in humans has been implemented in clinical practice. In a previous study, we demonstrated that estimation of renal perfusion based on a single positron emission tomography/computed tomography (PET/CT) scan with Rubidium-82 (^82^Rb) is feasible and found an approximate 5% intra-assay coefficient of variation for both kidneys, indicative of a precise method.This study’s aim was to determine the day-to day variation of ^82^Rb PET/CT and to test the method’s ability to detect increased RBF induced by infusion of amino acids.

**Methods:**

Seventeen healthy subjects underwent three dynamic ^82^Rb PET/CT scans over two examination days comprising: Day A, a single 8-minute dynamic scan and Day B, two scans performed before (baseline) and after RBF stimulation by a 2-hour amino acid-infusion. The order of examination days was determined by randomization. Time activity curves for arterial and renal activity with a 1-tissue compartment model were used for flow estimation; the K_1_ kinetic parameter representing renal ^82^Rb clearance. Day-to-day variation was calculated based on the difference between the unstimulated K_1_ values on Day A and Day B and paired t-testing was performed to compare K_1_ values at baseline and after RBF stimulation on Day B.

**Results:**

Day-to-day variation was observed to be 5.5% for the right kidney and 6.0% for the left kidney (n = 15 quality accepted scans). K_1_ values determined after amino acid-infusion were significantly higher than pre-infusion values (n = 17, p = 0.001). The mean percentage change in K_1_ from baseline was 13.2 ± 12.9% (range − 10.4 to 35.5) for the right kidney; 12.9 ± 13.2% (range − 15.7 to 35.3) for the left kidney.

**Conclusion:**

Day-to-day variation is acceptably low. A significant K_1_ increase from baseline is detected after application of a known RBF stimulus, indicating that ^82^Rb PET/CT scanning can provide a precise method for evaluation of RBF and it is able to determine changes herein.

**Clinical Trial Registration:**

EU Clinical Trials Register, 2017-005008-88. Registered 18/01/2018.

## Introduction

RBF reduction is believed to play an important role in the pathogenesis of two major contributors to the overall global disease burden: Hypertension and renal disease [1–5].

Clearance measurements based on blood- and urine sampling, as well as imaging techniques such as CT, magnetic resonance and ultrasonography are currently available methods for determining renal plasma flow (RPF) and/or RBF in humans. However, implementation of these techniques into routine clinical practice has been largely halted, as they have been shown to be more or less cumbersome and as having uncertainties associated with renal perfusion determination [6–10]. Development of a more practical, non-invasive, clinical method for estimation of renal perfusion, which is able to measure significant perfusion changes, is still in demand.

Organ perfusion determination based on PET and the use of perfusion tracers has been used clinically for evaluation of brain and myocardial perfusion for decades. For renal perfusion imaging, the tracers nitrogen-13-labeled ammonia and oxygen-15 labelled water have been used [11, 12]. However, use of these tracers in the clinical setting is challenging since their use is strictly limited to centers with on-site cyclotron access. In contrast, ^82^Rb, a monovalent cationic analog of potassium with a short physical half-life of 75 s, is widely used for clinical myocardial perfusion imaging using PET/CT scanning [13–15]; as such, ^82^Rb is readily available at clinical sites performing this study.

Initial human and method feasibility studies using pharmacokinetic analysis to provide flow estimation, have shown the human kidneys to have a high perfusion rate and homogeneity, as well as a high ^82^Rb first pass extraction, uptake and renal accumulation, and as such, they are highly suitable for RBF dynamic PET studies [16–19] .

**Fig. 1:**
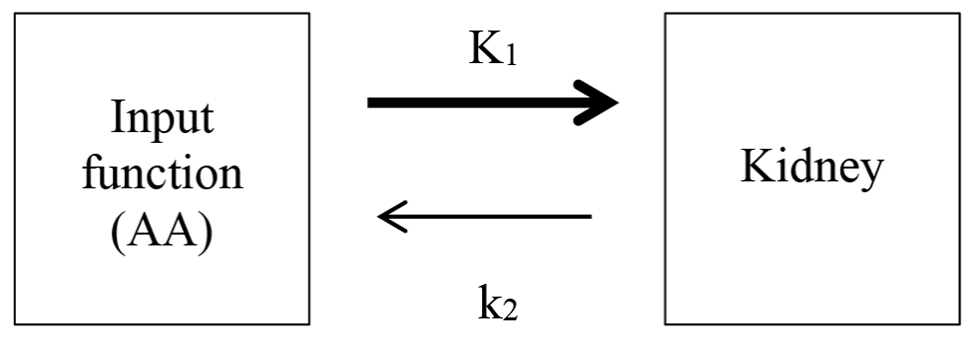
1-tissue compartment model used for estimation of flow. K_1_ is the uptake rate from blood into tissue; k_2_ is the washout rate from tissue into blood. No discernible ^82^Rb activity has been observed in the urine [18, 19]

In a previous proof-of-method study [19], we have demonstrated the feasibility of measuring ^82^Rb clearance using the abdominal aorta (AA) as the image-derived input function (IDIF) to a 1-tissue compartment model (Fig. 1), and shown the method to have a low intra-assay coefficient of variation and excellent inter-observer reliability. This study also suggested an uncertainty in the physiological interpretation of what ^82^Rb PET/CT clearance measurements actually represent: RBF, as is the current interpretation, or is the method in fact providing an estimate of the effective renal plasma flow (ERPF) [19]? Regardless of this uncertainty, ^82^Rb PET/CT does provide a measure of renal clearance and further studies of the method’s accuracy, precision and its ability to detect genuine renal flow changes are required before its possible clinical implementation.

In healthy subjects the normal renal hemodynamic response to protein loading is an increase in RPF and glomerular filtration rate (GFR), with increases above the unstimulated state known as the renal functional reserve. Increments in RPF are seen to rise by 7–27% after intravenous amino acid infusion [20–22]. In healthy middle-aged and elderly subjects, the renal functional reserve is preserved, but decreases with age [23–25].

The aim of this study was to test the ability of ^82^Rb PET/CT to detect genuine differences in flow: firstly, we determined the method’s day-to-day variation and secondly, we applied a known RPF stimulus to test its ability to detect increased flow.

## Materials and methods

### Study design

**Fig. 2:**
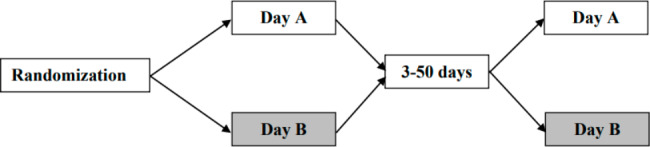
Study design

The study was a randomized cross-over interventional study (Fig. 2). Each subject underwent three 8-minute dynamic ^82^Rb PET/CT scans over two days: Day A and Day B (mean interval 9.7 ± 5.0 days between examination days). On Day A, a single scan was performed; on Day B, a scan was performed before (baseline) and after a 2-hour sustained amino acid-infusion. The order of examination days was allocated by computer-generated randomization in blocks of four.

### Participants

Healthy subjects were recruited in the period September 2018 to February 2019 by advertising in the local newspaper. Each participant completed a screening program prior to enrollment consisting of a medical history, clinical examination, electrocardiography, office blood pressure measurement, urine dip stick and the following blood samples: sodium, potassium, creatinine, albumin, alanine aminotransferase, hemoglobin, erythrocyte volume fraction, leucocytes, and thrombocytes. Pregnancy was ruled out in fertile female subjects.

Inclusion criteria were men and women aged 50 to 80 years with a body mass index (BMI) in the range 18.5 to 30.0 kg/m^2^. Fertile women had to use either hormonal contraceptives or exert sexual abstinence during the entire study period. Exclusion criteria were current use of medicine except contraception, pregnancy or breastfeeding, alcohol intake > 14 units per week for men and > 7 units per week for women, smoking, substance abuse, arterial hypertension, current malignant disease, or signs of clinically relevant renal disease, heart disease, pulmonary disease, liver disease, endocrine disease or neurological disease in the medical history, clinical examination or in the screening tests. Withdrawal criteria were development of one or more exclusion criteria or withdrawal of consent.

### Number of subjects

To ensure a power of 80%, significance level of 5%, minimal relevant difference in RBF of 0.11 ml/min/cm^3^ and a standard deviation (SD) of the difference between two values for the same subject of 0.12 ml/min/cm^3^, 12 subjects were required for the study. To account for expected dropout, 20 subjects were included.

### Pre-scan procedure

In the 24 h preceding each examination day, standardization of fluid intake to 35 ml/kg body weight plain water was implemented, no dietary restrictions were imposed (none of the subjects were on a vegetarian diet) and subjects were instructed to avoid strenuous exercise. On scan days, subjects arrived at 8 am at the Department of Nuclear Medicine, Regional Hospital Herning, Denmark, after a fasting period of 12 h. No fluid intake was allowed during the fast.

### Radiopharmaceutical (study drug)

^82^Rb is acquired for each individual dynamic study scan by elution of a Strontium-82 (^82^Sr)/^82^Rb generator (Cardiogen-82; Bracco Diagnostics Inc., Monroe Township, NJ, USA). Necessary quality control procedures were performed on each examination day according to approved guidelines (Bracco Diagnostics Inc.), including tests for ^82^Sr and ^85^Sr breakthrough. The system was calibrated to deliver a dose of 555 Megabecquerel (MBq) ^82^Rb for each injection.

### PET/CT-scan

PET/CT scans were carried out on a single PET/CT scanner (Siemens Biograph mCT; 64 slice-4R) with an axial field-of-view (FOV) of 22 cm. The PET/CT scanner was quality controlled and calibrated according to necessary system procedures on each examination day.

On Day A, a peripheral venous catheter was inserted into a cubital vein for ^82^Rb injection. On Day B, an additional catheter was placed in a cubital vein in the other arm for amino acid-infusion. Subjects rested in a sitting position for about 30 min before voiding. They were then placed in the PET/CT scanner in the supine position with arms above the head and the generator infusion system coupled to the catheter on the left side, after which the subjects underwent a single bed-position PET/CT scan with the AA and the entire kidneys in the same FOV. To position the scanner over the required FOV, an initial scout image was performed followed by a low-dose CT scan for attenuation correction purposes only (25 mAs, 100 kV). Immediately hereafter, a 555 MBq ^82^Rb bolus injection was administered and a dynamic list-mode PET scan acquired for 8 min [18, 19]. On Day A, the cubital catheter was removed after completion of the scan. On Day B, an intravenous infusion of amino acids was started after the first scan. Subjects rested in a sitting position for the duration of the infusion. The cubital catheters were removed after completion of a second, identical, PET/CT scan which was performed after 120 min of amino acid infusion.

List mode data were rebinned using 37 frames (20 × 3 s, 10 × 12 s, 4 × 30 s and 3 × 60 s) and iteratively reconstructed (21 subsets, 2 iterations) using Siemens TrueX and time-of-flight reconstruction in a 256 × 256 matrix (3.2 × 3.2 × 3.0 mm) and post-filtered with a 5.0 mm Gaussian filter, resulting in attenuation-corrected and decay-corrected dynamic sequences. We did not find it necessary to adjust for motion of the kidneys.

Each participant received a total effective radiation dose < 3.5 milli-Sievert (mSv): Low-dose CT scans contributing 1.2 mSv and each ^82^Rb bolus injection contributing 1.26 µSv/MBq.

### Amino acid-infusion

The amino acid-solution consisted of a mixture of essential and non-essential amino acids (Vamin 18 g N/l, electrolyte free, 1130 mosm/L, Fresenius Kabi AB, Sweden), as described in Table 1. Amino acids were infused with a rate of 0.029 ml/kg/min for a duration of 120 min [26].

**Table 1:** Composition of amino acids (g/L) in Vamin 18 g N/l, electrolyte free, 1130 mosm/L

L-alanine	16.0	L-leucine	7.9
L-arginine	11.3	L-lycine	9.0
L- aspartic acid	3.4	L-methionine	5.6
L-cysteine (+ L-cystine)	0.56	L-phenylalanine	7.9
L- glutamic acid	5.6	L-proline	6.8
Glycine	7.9	L-serine	4.5
L-histidine	6.8	L-threonine	5.6
L-isoleucine	5.6	L-tryptophan	1.9

### Analysis of ^82^Rb PET/CT studies

PMOD software (PMOD Technologies Ltd., Zurich, Switzerland, version 4.102) was used to perform pharmacokinetic modelling.

**Fig. 3:**
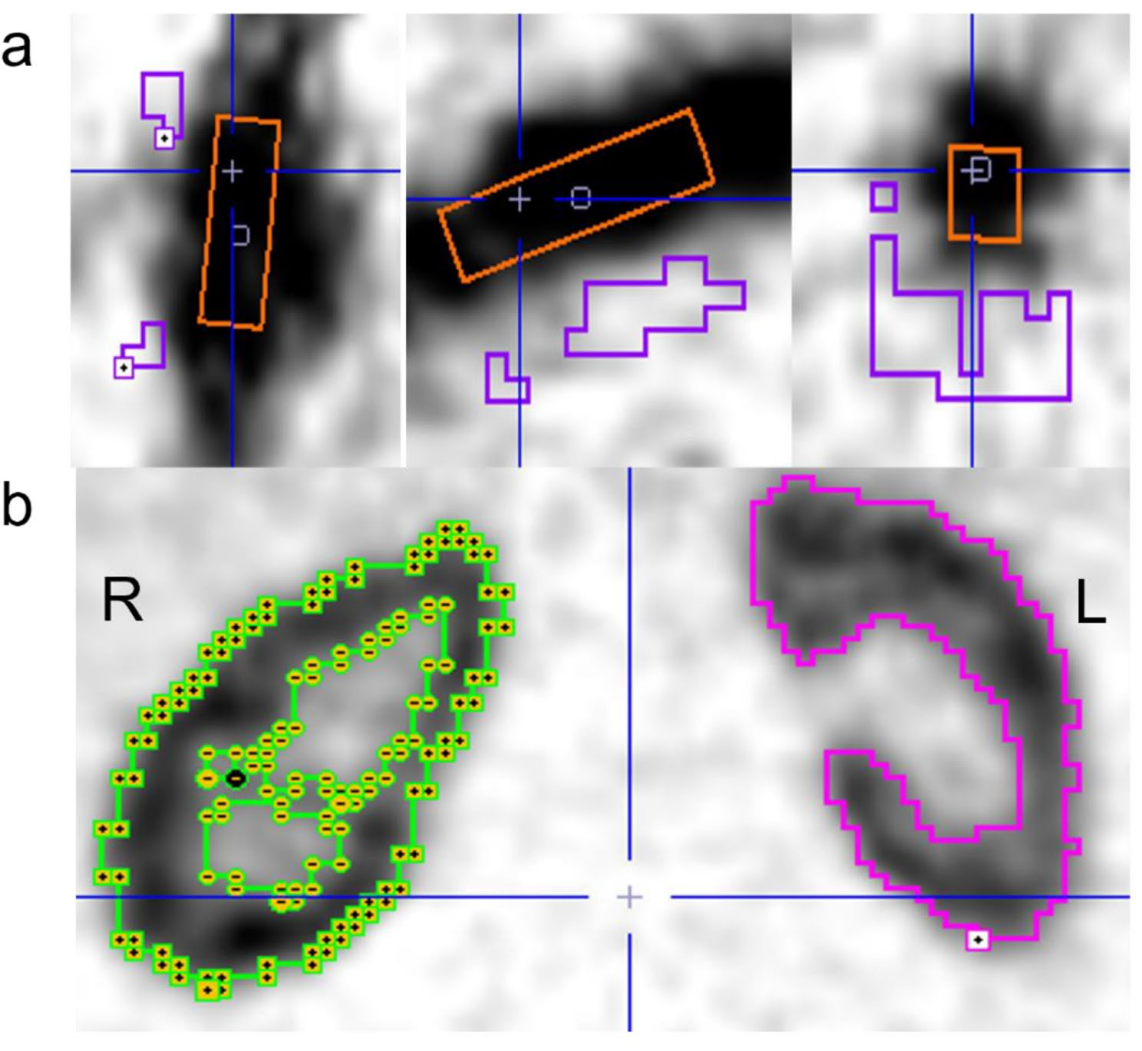
VOIs placed in the AA (orange) and aortic background (purple) (a) and contouring the kidneys in one study subject (b). R = right. L = left

Data analysis was performed as described in our preceding study [19] with the following minor adjustments: Volumes of interest (VOIs) were defined for the AA and the kidneys (Fig. 3) with AA box-dimensions 10 × 10 × 40 mm^3^. To define the aortic background and kidney-VOIs, limiting boxes were placed around the organs-of-interest and the cold contour tool (typical cut-off 20–35%) and hot contour tool (typical cut-off 50–55%) used for the respective VOIs. The deliminated kidney-VOIs represent the total renal volume, including both the cortex and the medulla but excluding the renal pelvis; discrimination of the cortex and medulla was not possible.

Time activity curves for each VOI were obtained to define the AA-derived input function for kinetic modelling and kidney tissue data. Necessary correction for partial volume effects and spill-over in the AA activity was performed using:

1 $${{\rm{R}}_{\rm{A}}}\left( {\rm{t}} \right) = \beta \times {{\rm{C}}_{\rm{A}}}\left( {\rm{t}} \right) + (1 - \beta ) \times {{\rm{C}}_{{\rm{Bg}}}}\left( {\rm{t}} \right),$$

where R_A_(t) is measured activity in the AA, C_A_(t) the corrected AA activity, β the required recovery coefficient (RC) (β = 0.612 as determined in phantom studies [19]), and C_Bg_(t) the measured aortic background activity. In contrast, kidneys-VOIs are so large that partial volume and spill-over effects are negligible, obviating the need for background correction. Kidney activity was however corrected for ^82^Rb count efficiency using a scanner specific RC of 0.659 determined by phantom studies (unpublished), and a blood volume fraction of 10% was assumed to account for activity from the vascular space within the kidney VOIs [27, 28].

For each kidney, K_1_ values (ml/min/cm^3^) were calculated using the 1-tissue compartment model (Fig. 1). The uptake rate K_1_ represents the renal ^82^Rb clearance (ml/min/cm^3^) [18, 29] and can, as previously described, be used as an estimate of flow [19] due to high first pass ^82^Rb extraction [16].

### Total flow estimation

Total renal ^82^Rb clearance can be estimated from the measured ^82^Rb clearance (K_1_) and the total kidney volume (V_Total_); the estimation of which, is given by the applied kidney-VOIs:

2 $${\text{Total}}\,{\text{renal}}\,{\text{clearance}} = {{\text{K}}_1} \times {{\text{V}}_{{\text{Total}}}}$$

Total ^82^Rb clearance was normalized to body surface area (BSA) using the Dubois formula [30]:

3 $$\text{B}\text{S}\text{A}=0.007184\times\text{h}\text{e}\text{i}\text{g}\text{h}\text{t}^{0.725}\times\text{w}\text{e}\text{i}\text{g}\text{h}\text{t}^{0.425}$$

Units of BSA are m^2^, with height (cm) and weight (kg).

### Statistical analysis

Statistical analysis was performed in SPSS Statistics ver. 20 (IBM Corp., Armonk, NY, USA).

A paired sample t-test was used for comparison of unstimulated K_1_ values on Day A and B and between K_1_ values for baseline and response to stimulation on Day B. Day-to-day variation was calculated based on the difference between the unstimulated K_1_ values on Day A and the unstimulated K_1_ values on Day B. Statistical significance was defined as p < 0.05.

## Results

### Demographics

**Fig. 4:**
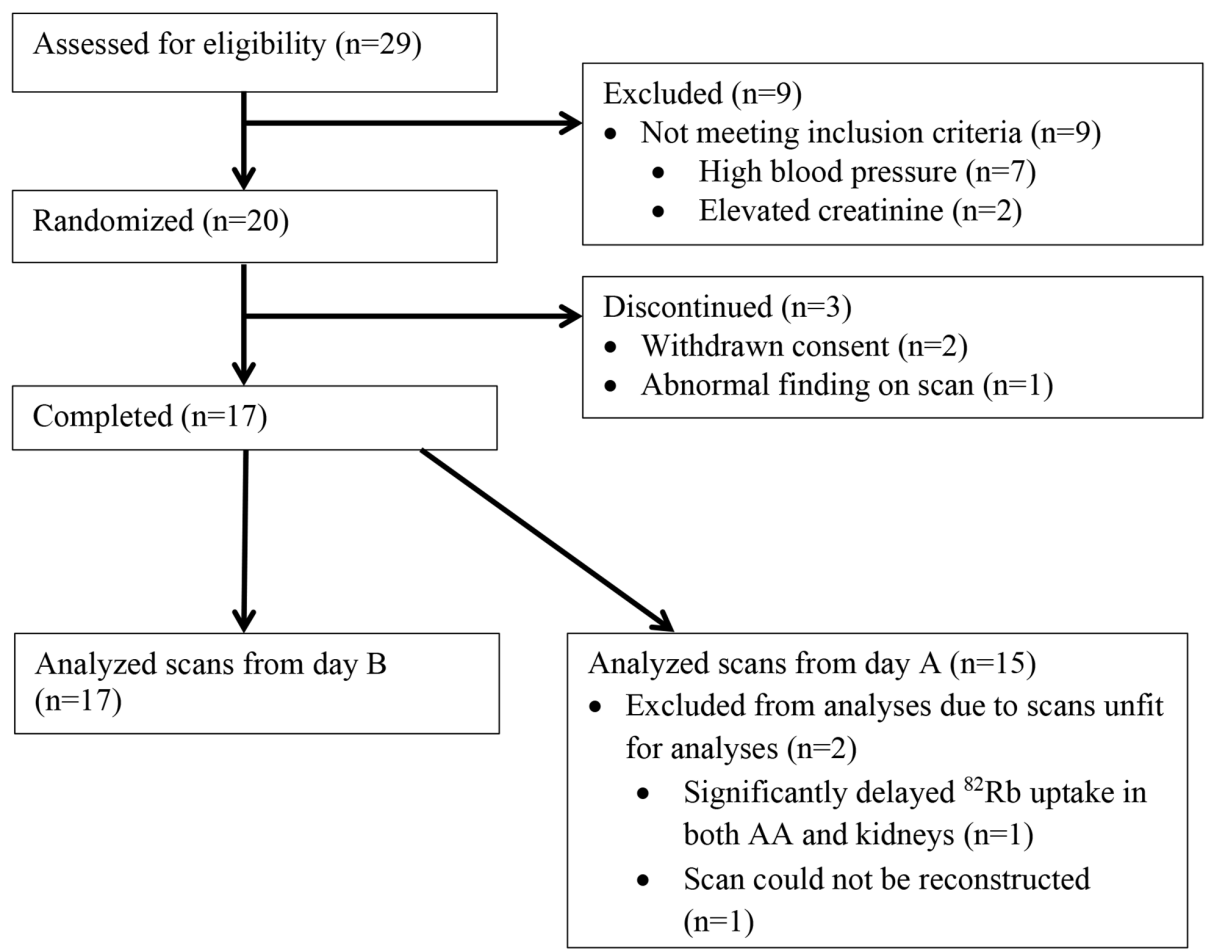
Subject flow in the study

Figure 4 illustrates the study’s participant flow. Twenty healthy subjects were included, and seventeen subjects completed the trial. For Day B, all completing subjects had quality accepted scans for analysis, but for Day A, two out of seventeen scans were rejected due to technical problems (Fig. 4). Clinical and biochemical characteristics of completing subjects are shown in Table 2.

**Table 2:** Clinical and biochemical characteristics (n = 17)

Age (years)	65 ± 6
Gender (women/men)	7/10
BMI (kg/m^2^)	24.1 ± 2.5
Systolic blood pressure (mmHg)	127 ± 8
Diastolic blood pressure (mmHg)	75 ± 6
Heart rate (beats/min)	70 ± 11
P-creatinine (µmol/L)	76 ± 11
eGFR_MDRD_ (mL/min/1,73m^2^)	84 ± 8
P-alanine aminotransferase (U/L)	23 ± 7
P-sodium (mmol/L)	141 ± 2
P-potassium (mmol/L)	3.8 ± 0.3
P-albumin (g/L)	42 ± 3
B-hemoglobin (mmol/L)	8.7 ± 0.7
B-leukocytes (x 109/L)	5.7 ± 1.5
B-thrombocytes (x 109/L)	243 ± 52
B-EVF	0.42 ± 0.03

BMI: body mass index; eGFR_MDRD_: estimated glomerular filtration rate calculated using the Modification of diet in renal Disease Study equation; EVF: erythrocyte volume fraction. Data are presented as mean ± SD.

### PET/CT scans

In all quality accepted scans, the FOV included AA and the entire kidneys with the following exception: In one subject, due to an unusual superior position of the right kidney just below the diaphragm, the cranial part of the kidney was omitted in the FOV of the first PET/CT scan for the Day B scan-series. Hereafter, for the second Day B-scan and on returning for Day A-scanning, it was ensured that the kidneys were included in the FOV in their entirety. As shown in our previous study, for normal, healthy kidneys, K_1_ determination for excluded cranial sections does not differ from global K_1_ for the entire kidney [19]. Hence, the K_1_ values determined from the truncated kidney scan are also included in the data analysis.

### Renal ^82^Rb clearance

**Fig. 5:**
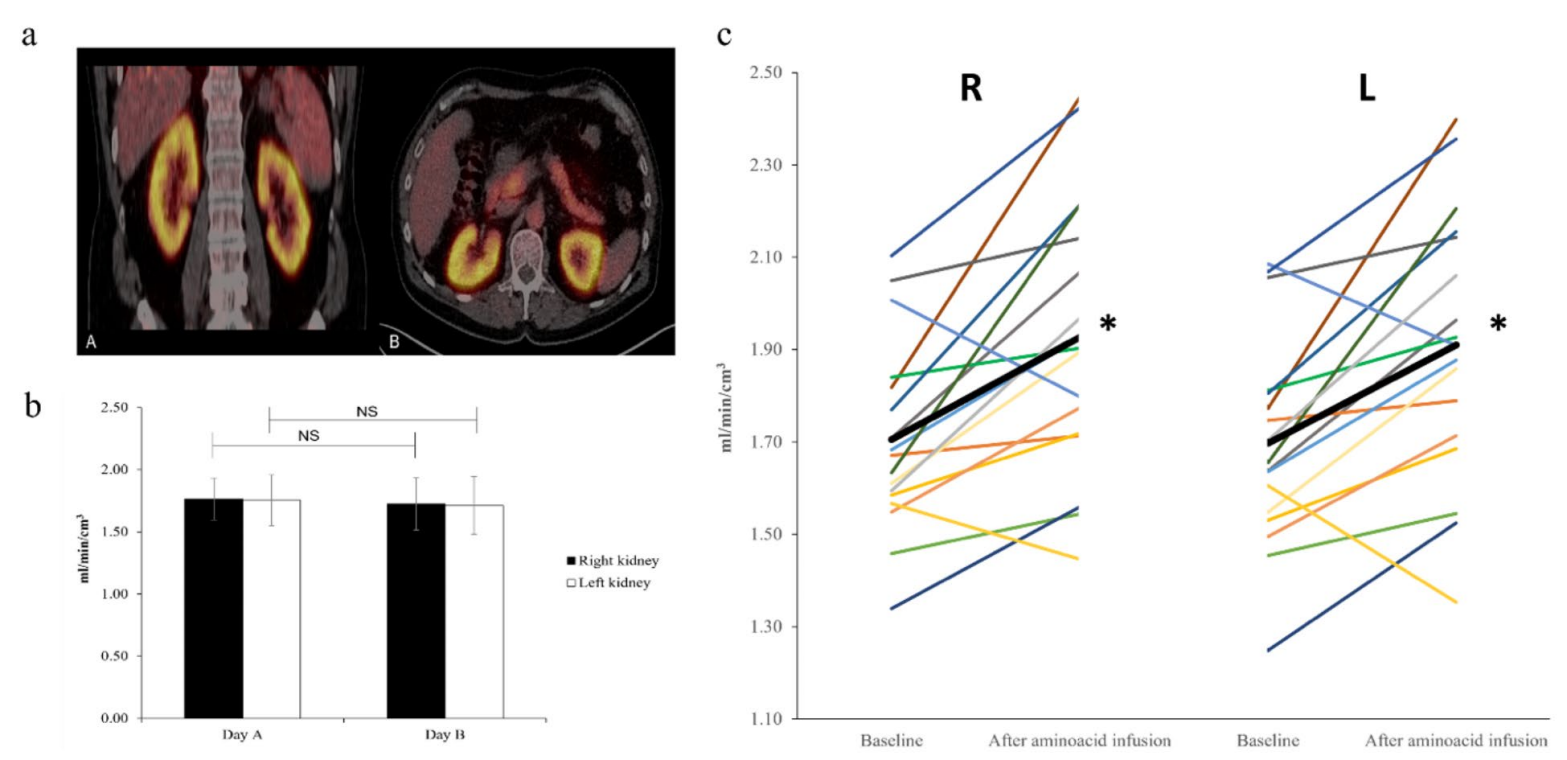
**a**: A typical example of ^82^Rb uptake in the kidneys during amino acid-infusion in one study subject. PET/CT images in the coronal plane (A) and horizontal plane (B). **b**: Mean unstimulated K_1_ values from Day A and B (baseline). Data are presented as mean ± SD. Paired t-test. NS: Non-significant. **c**: Individual K_1_ values at baseline and after amino acid-infusion for the right (R) and left kidney (L). The black bar illustrates the mean. Paired t-test *: p = 0.001

High renal ^82^Rb uptake was confirmed in middle-aged healthy subjects (Fig. 5a); both for unstimulated and stimulated RBF.

No significant difference was observed between unstimulated K_1_ values determined on Day A and Day B (Fig. 5b), with day-to-day variation found to be 5.5% for the right kidney and 6.0% for the left kidney.

K_1_ values at baseline and in response to amino acid-infusion for each individual are displayed in Fig. 5c. For 15 out of 17 subjects, the response to amino acid loading was an increase in K_1_, whereas in two subjects, K_1_ decreased.

Average post-infusion values were significantly higher than average baseline values for both kidneys (p = 0.001, right kidney and p = 0.001, left kidney) (Fig. 5c), with the mean relative change in K_1_ being 13.2 ± 12.9% (range − 10.4 to 35.5) for the right kidney; 12.9 ± 13.2% (range − 15.7 to 35.3) for the left kidney. No significant difference was observed between right and left kidneys for either the unstimulated or stimulated K_1_ values.

Calculated total renal flow values are summarized in Table 3: BSA normalized unstimulated renal flow was estimated to be 454 ± 83 ml/min/1.73 m^2^ and BSA normalized stimulated renal flow 522 ± 109 ml/min/1.73 m^2^. For the subject with truncated kidney data, kidney volumes and hence total flow values would be underestimated; thus, these are omitted from this evaluation.

**Table 3:** Estimation of total renal ^82^Rb clearance

	Average K_1_ (ml/min/cm^3^)	Total renal volume (cm^3^)	Total renal flow (ml/min)
Unstimulated state* (n = 14)	1.75 ± 0.19	277 ± 30	486 ± 87
Stimulated state (n = 16)	1.92 ± 0.30	290 ± 34	556 ± 102
Young adults** Unstimulated state (n = 18)	2.80 ± 0.43	296 ± 30	825 ± 122

Data are presented as mean ± SD. *Calculation basis: Mean of unstimulated K_1_ flow values from Day A and Day B (baseline). **Data from young adults from [19]. Total renal volume is estimated from the kidney VOIs in PMOD.

## Discussion

In a previous study, we have confirmed the feasibility and precision of ^82^Rb PET/CT for estimation of renal perfusion using the AA as an IDIF in a 1-tissue compartment model [19]. This study further validates the possibility of using this imaging method for clinical renal flow estimation, by showing the day-to-day variation in ^82^Rb clearance is small enough (~ 6%) to allow for genuine observation of the expected, significant, increase in flow values compared with baseline values for both kidneys when a known RBF stimulus is applied.

### Method precision and day-to-day variation

The magnitude of the determined day-to-day variation associated with ^82^Rb PET/CT renal clearance estimation is a combination of the physiological RBF daily variation and the PET/CT method’s inherent measurement precision, the latter estimated by the inter-assay coefficient of variation from repeated measurements of the same subject. As such, day-to-day variation is expected to be the (slightly) larger of the two quantities. We have previously determined the method’s intra-assay coefficient of variation to be low; ~5% for both kidneys [19]. In combination with a ~ 6% value of day-to-day variation, which is highly acceptable, these measurement uncertainty values suggest ^82^Rb PET/CT to be a precise method for evaluation of renal clearance.

### Renal functional reserve

Our results confirm that renal functional reserve is preserved in healthy middle-aged subjects.

For both kidneys, a significant increase in ^82^Rb clearance was detected in response to a 2-hour long amino acid-loading, compared to pre-infusion values. Although RBF is subject to circadian variation, with significant differences having been demonstrated in the afternoon and at night [31, 32], the ^82^Rb clearance increase found in this study cannot merely be attributed to this cyclic variation: Pre- and post-infusion values were measured with only a 2 h time-lapse between measurements pre-noon.

The mean ^82^Rb clearance increase was determined to be ~ 13% for both kidneys, which is in agreement with findings from several other studies reporting flow increments in the range 7 − 15% when using an identical amino acid solution, infusion rate and similar infusion durations [21, 22, 26, 33]. Similarly, individual renal hemodynamic responses to amino acid-infusion, show that not all subjects responded with an increase in renal perfusion, which is in full agreement with results presented in similar studies of amino acid induced changes in renal hemodynamics [21, 22, 33].

Considering that renal functional reserve has been shown to decline with age [23–25], and despite the mean age of this study’s subjects being considerably older (65 years) than those in other studies (median age: 31–39 years), the subjects in this study exhibit a quite high flow increase. This however is not unheard of, as other authors have reported considerably higher RBF increments (18 -25%) after vasodilatory stimulus in middle-aged subjects where a simultaneous infusion of amino acids and dopamine was used to elicit a maximal vasodilatory stimulus. This can explain the higher RBF rise published in the literature compared to the results of this study [23, 25].

### Method reliability

The capability of ^82^Rb PET/CT to detect an expected significant amino acid-induced RBF increase, which in magnitude, agrees with previous findings, supports the reliability of ^82^Rb PET/CT for clinical RBF determination. Compared to the previously measured 5% intra-assay coefficient of variation [19] and the physiological 6% day-to-day variation for the method, the observed increase in clearance appears significant and not just a result of biological and measurement uncertainties.

### K_1_ - age dependence

Both RBF and GFR decrease with age; RBF proportionally more than GFR by approximately 10% per decade [34]. In our recent proof-of-method-study [19], 18 healthy adults with a younger mean age of 21 ± 4 (range 18 to 36) years were scanned using ^82^Rb PET/CT and their renal clearance estimated. Here the imaging scans and K_1_ analyses were performed using almost identical conditions as for this current study, the only minor differences being a 4-hour shorter fasting period prior to scan-acquisition and the minor adjustments to the PET/CT data analysis as described in the Methods section. Furthermore, analysis of the PET/CT data was performed by the same observer in both studies. These minor differences are not considered to influence the results significantly between the two studies and thus, the unstimulated K_1_ values from this present study are comparable with unstimulated K_1_ values determined previously, the only major difference being the ages of the participants.

The mean age difference between the two studies was 44 years. In comparison, the middle-aged adults in this study had substantially lower ^82^Rb clearance and total flow values than those observed for the young adults of the former study; estimated total ^82^Rb clearance for middle-aged adults being ~ 60% of the young adults (Table 3). This supports the expectation that RBF falls with age and that ^82^Rb PET/CT shows reliable behavior in RBF determination.

### Method accuracy

Accuracy assessment of ^82^Rb PET/CT for RBF estimation is not possible under the study design of this paper. Additionally, due to open questions regarding what it is ^82^Rb PET/CT is measuring physiologically, the validation of method accuracy is a non-trivial question. To validate any method, measurement data needs to be referred to a standard, relevant to the quantity being observed. If ^82^Rb PET/CT estimates RBF, as existing literature assumes [18, 35], the ideal reference method would be oxygen-15 labelled water PET/CT studies [36]. However, as discussed in our previous study [19], there is some uncertainty to whether ^82^Rb PET/CT is actually a measurement of RBF, or in fact, ERPF. The latter based on the indication that ^82^Rb is primarily present in the plasma during the short duration of the dynamic PET/CT study [37, 38], as well as the observation that our previously measured ^82^Rb clearance values (766 ± 114 ml/min/1.73 m^2^) [19] are similar to historically published values for ERPF [26, 39, 40]. If ^82^Rb PET/CT is actually a measure of ERPF, then other reference methods such as para-aminohippurate (PAH)- and iodohippurate (OIH) clearance methods become the relevant standards to compare with. Here, there is the added complication that the extraction coefficient is unknown in humans.

Additionally, there are several areas of bias in determining absolute quantitative values e.g. the assumed vascular volume in the tissue, time delays between ^82^Rb appearing earlier in the abdominal aorta than in the kidneys, partial volume effects – especially in the relative fractions of cortex and medulla tissue in the deliminated VOIs, as well as kidney movement due to breathing; all issues needing to be addressed and, if possible, corrected for when implementing the quantitative analysis. Thus, obtained perfusion estimates can only be expected to correlate to renal perfusion and not give an absolute value. However, even though the accuracy question remains unanswered, this does not negate the ^82^Rb PET/CT method being used to address relative renal clearance measurement in patients, before and after interventions.

### Study strengths and limitations

The major strengths of this study are the combination of its randomized design and the standardization of pre-scan conditions regarding fluid intake, exercise level and duration of fasting. The study population consisted of healthy adults and as such measured results were uninfluenced by medications. The cross-over design did not include an infusion intervention on Day A, which might be considered a study limitation; however, since the study aim was to evaluate if a known amino acid stimulus could be detected as a rise in RBF using ^82^Rb PET/CT, and not to investigate the effect of amino acid-loading on RBF, an equivalent intervention on Day A was not required to fulfill the purpose of the study; it would result in unnecessary radiation dose to the participants. Due to the strict screening procedure prior to enrollment, the study population was homogenous and healthy, thus, additional feasibility studies for ERPF/RBF determination are warranted in subjects suffering from hypertension or kidney disease.

## Conclusion

To our knowledge this study is the first to address the reliability of the ^82^Rb PET/CT method for evaluation of renal perfusion. Day-to-day variation is low at ~ 6% for both kidneys and application of a standard RPF stimulus resulted in a significant increase in the observed ^82^Rb clearance from baseline values for both kidneys as expected; the mean percentage change being ~ 13% for both kidneys. Additionally, the expected decrease in renal perfusion with age is also observed, indicating that ^82^Rb PET/CT is a precise and reliable method for analysis of relative changes in renal perfusion.

**Declarations**.

## Data Availability

The datasets and trial protocol (Danish) are available from the corresponding author on reasonable request.

## Abbreviations

AA: abdominal aorta
BMI: Body mass index
BSA: Body surface area
CT: Computed tomography
ERPF: Effective renal plasma flow
FOV: Field of view
GFR: Glomerular filtration rate
IDIF: Image-derived input function
MBq: Megabecquerel
mSv: milli-Sievert
PAH: para-aminohippurate
OIH: iodohippurate
PET: Positron emission tomography
^82^Rb: Rubidium-82
RBF: Renal blood flow
RPF: Renal plasma flow
^82^Sr: Strontium-82
SD: Standard deviation
VOI: volume of interest
